# The Dangers of Inflating the Stomach

**Published:** 1904-01-16

**Authors:** 


### THE DANGERS OF INFLATING THE STOMACH.

Inflation of the stomach is such a valuable aid in the examination of patients who present themselves with certain gastric complaints that a consideration of the methods of carrying it out may be worthy of attention. It is usual to distend the stomach in one of two ways: (1) by the administration of drugs which evolve $CO_2$ gas when taken into the stomach; (2) by passing a stomach tube and pumping in air till sufficient dilatation has been effected. The latter method has the great advantage of allowing the amount of inflation to be under control, and by permitting the patient to pump in the air himself pain from over-distension may be avoided. But there is the drawback that a stomach tube has to be passed. This is a difficulty that has been unduly magnified. Indeed it would appear that the fear of the stomach tube is often as much on the side of the medical man as the patient. Tact, gentleness, and patience will usually overcome the scruples of the latter. Against the more common method of giving a dose of sodium bicarbonate followed up by a solution of tartaric acid is the circumstance that no regulation of the amount of distension is possible, and consequently much discomfort and even danger may be caused. Dr. M. Behrend[1] reports three cases in which he believes that death was directly due to inflation of the stomach by the method in question. In one of these patients, a woman of 68, great distress was felt immediately after the effervescing powders had been taken. Half an hour later she vomited a large quantity of blood, and hæmatemesis continued till her death, 20 hours after the administration. The two other patients were men, and in both there was malignant disease of the lower end of the œsophagus. Consequently the evolution of $CO_2$ gas took place in the œsophagus. In both of these instances the immediate result was great pain followed by prostration and death. In view of these three cases it will be advisable to exercise a good deal of caution before employing the $CO_2$ method in the future, and the more so as it is quite probable that other disasters have occurred from its use which have not been recorded in the medical press.

[1] Medical News, Dec. 19, 1903.

